# Supplementary material for: Evaluating changes in the prevalence of female genital mutilation/cutting among 0-14 years old girls in Nigeria using data from multiple surveys: A novel Bayesian hierarchical spatio-temporal model
Source: PLoS One. 2021 Feb 12;16(2):e0246661. doi: 10.1371/journal.pone.0246661 (PMC7880428; doi:10.1371/journal.pone.0246661)
Supplement: Attachment — Submitted filename: comments to authors.docx [file pone.0246661.s001.docx]

**General comments to authors**

1. What are the reasons the different year data set combined? Because six years data are variation from year to year.
2. In table-3 Please clearly explain the variation between FGM women and FGM in the variable names.
3. In table -3 The respondent’s household wealth index categories, what means second?
4. Revised the table-3 because, it has not full output for example girls age p-value etc.
5. There is a missing value. How to handle these missing values? please describe the algorithm.
6. How the authors choose the prior distribution and a=1 and b=0.005?
7. Why not use informative prior distribution? since there are six years data, then the past five years are prior empirical evidence.
8. Please separate the discussion and conclusion in this paper.
