# Supplementary material for: Evaluating changes in the prevalence of female genital mutilation/cutting among 0-14 years old girls in Nigeria using data from multiple surveys: A novel Bayesian hierarchical spatio-temporal model
Source: PLoS One. 2021 Feb 12;16(2):e0246661. doi: 10.1371/journal.pone.0246661 (PMC7880428; doi:10.1371/journal.pone.0246661)
Supplement: Attachment — Submitted filename: Response to Reviewers.docx [file pone.0246661.s002.docx]

22^nd^ December 2020.

Dear Editor,

Dr Kannan Navaneetham

Academic Editor,

PLOS ONE.

**RE: PONE-D-20-16745- Evaluating changes in the prevalence of female genital mutilation/cutting among 0-14 years old girls in Nigeria using data from multiple surveys: A novel Bayesian hierarchical spatio-temporal model**

We the authors of the above-mentioned paper would like to write this rebuttal letter in response to the questions/comments made by reviewers and the Editor. First, we would like to thank you for your immense efforts, commitments and very constructive comments aimed at improving the quality of the manuscript.

In response to the question/comments, we are pleased to say that we have effected all necessary changes and corrections, and these could easily be seen in one of the attached documents named ‘Revised Manuscript with Track Changes’. As advised, we have also attached a separate document named ‘Manuscript’ which do not have track changes.

For ease of exposition, we have provided on a point-by-point basis response to each of the questions/comments/points in the ‘General comments to authors’ as detailed below. Responses were also provided to other comments including those made with respect to image copyrights and we hope that they were all satisfactorily addressed.

Please do not hesitate to get back to me if there is any need to do so.

Yours Sincerely,

Chibuzor Christopher Nnanatu, PhD, MSc, BSc

[Chibuzor.nnanatu@northumbria.ac.uk](mailto:Chibuzor.nnanatu@northumbria.ac.uk)

*Dear Dr. Nnanatu,*

*Thank you for submitting your manuscript to PLOS ONE. After careful consideration, we feel that it has merit but does not fully meet PLOS ONE’s publication criteria as it currently stands. Therefore, we invite you to submit a revised version of the manuscript that addresses the points raised during the review process.*

*Please submit your revised manuscript by Jan 17 2021 11:59PM. If you will need more time than this to complete your revisions, please reply to this message or contact the journal office at plosone@plos.org. When you're ready to submit your revision, log on to https://www.editorialmanager.com/pone/ and select the 'Submissions Needing Revision' folder to locate your manuscript file.*

*Please include the following items when submitting your revised manuscript:*

*A rebuttal letter that responds to each point raised by the academic editor and reviewer(s). You should upload this letter as a separate file labeled 'Response to Reviewers'.*

*A marked-up copy of your manuscript that highlights changes made to the original version. You should upload this as a separate file labeled 'Revised Manuscript with Track Changes'.*

*An unmarked version of your revised paper without tracked changes. You should upload this as a separate file labeled 'Manuscript'.*

*If you would like to make changes to your financial disclosure, please include your updated statement in your cover letter. Guidelines for resubmitting your figure files are available below the reviewer comments at the end of this letter.*

*If applicable, we recommend that you deposit your laboratory protocols in protocols.io to enhance the reproducibility of your results. Protocols.io assigns your protocol its own identifier (DOI) so that it can be cited independently in the future. For instructions see: http://journals.plos.org/plosone/s/submission-guidelines#loc-laboratory-protocols*

*We look forward to receiving your revised manuscript.*

*Kind regards,*

*Kannan Navaneetham, PhD*

*Academic Editor*

*PLOS ONE*

**General comments to authors**

1. What are the reasons the different year data set combined? Because six years data are variation from year to year.

**Response:** Thank you. The different years datasets were combined to simultaneously investigate spatial and temporal trends in the practice of female genital mutilation/cutting (FGM/C) across the Nigeria 36 states and the federal capital territory (FCT). In addition, by combining the various datasets, we gained more statistical power to better estimate parameters with higher precision.

2. In table-3 Please clearly explain the variation between FGM women and FGM in the variable names.

**Response:** Thank you. We used $fgm_{woman}$ to denote the FGM/C status of a girl’s mother which takes the value ‘cut’ if mother has been circumcised and ‘uncut’, otherwise. While $fgm$ (without the subscript ‘woman’) is used to denote the response variable- a girl’s FGM/C status which is binary and takes the value of 1 if cut and 0 if uncut. We have updated the main manuscript where the two variables may not have been clearly distinguished specifically in Table 1 and equation 3.

3. In table -3 The respondent’s household wealth index categories, what means second?

**Response:** Thank you. For the respondent’s wealth quintile, the ‘second’ level is equivalent to having ‘lower’ wealth index which is second to the poorest. Thus, households classified as being in the second quintile are only *richer* than the ‘lowest’.

4. Revised the table-3 because, it has not full output for example girls age p-value etc.

**Response:** Thank you. We have now updated Table 3 to ensure that the available values are correctly entered while the empty cells due to unavailability of data are marked accordingly.

5. There is a missing value. How to handle these missing values? please describe the algorithm.

**Response:** Thank you. As stated in the main manuscript, two statistical software were utilized for the various analyses. With respect to handling missing values, the bivariate descriptive analysis using Stata and the Bayesian geo-additive multilevel regression models using R employed listwise deletion algorithm for observations with missing values. Note that women included in the analysis were only those with living daughters aged 0-14 years old and those who were able to provide information on their daughters’ FGM/C statuses.

6. How the authors choose the prior distribution and a=1 and b=0.005?

**Response:** Thank you. The hyperprior values for the variance parameters $\tau_{j}\sim InverseGamma(a=1,b=0.0005)$ were chosen following sensitivity analysis using various hyperprior choices including that of the default setting of R2BayesX (a=b=0.001). Convergence diagnostics relied on trace plots (sample paths plot), autocorrelation function (ACF) function plots and kernel density plots. While model choice relied on the Deviance information criterion (DIC).

7. Why not use informative prior distribution? since there are six years data, then the past five years are prior empirical evidence.

**Response:** Thank you. Yes, agreed that we could draw on the knowledge from the previous five years data for an informative prior distribution, for example, we could assign informative multivariate Gaussian priors to the fixed effects parameters instead of the diffuse priors ($\pi\left( \gamma\right)\propto constant$). However, such informative priors are not yet supported by BayesX. Nevertheless, the various prior choices utilized have been found to work well (e.g., Brezger, Kneib & Lang, 2005).

8. Please separate the discussion and conclusion in this paper.

**Response:** Thank you. We have now separated the discussion and conclusion into two distinct sections in the main manuscript. The conclusion section is found immediately after discussion.

**References**

Brezger, A., Kneib, T. and Lang, S. (2005). BayesX: Analyzing Bayesian Structured Additive Regression Models. *Journal of Statistical Software*, **14**(11).

Journal Requirements:

When submitting your revision, we need you to address these additional requirements.

**Response:** Thank you

1. Please ensure that your manuscript meets PLOS ONE's style requirements, including those for file naming. The PLOS ONE style templates can be found at

https://journals.plos.org/plosone/s/file?id=wjVg/PLOSOne_formatting_sample_main_body.pdf and

https://journals.plos.org/plosone/s/file?id=ba62/PLOSOne_formatting_sample_title_authors_affiliations.pdf

**Response:** Thank you

2. Please ensure that you in your Methods section report the question used to derive data (both outcome variables and explanatory variables), and specify how variables were defined and categorised. Please also ensure that you discuss missing data and imputation.

**Response:** Thank you. We believe that these have been sufficiently captured in the ‘Methods’ Section.

3.We note that [Figure(s) 1, 3, 4, 7 and 8] in your submission contain map/satellite images which may be copyrighted. All PLOS content is published under the Creative Commons Attribution License (CC BY 4.0), which means that the manuscript, images, and Supporting Information files will be freely available online, and any third party is permitted to access, download, copy, distribute, and use these materials in any way, even commercially, with proper attribution. For these reasons, we cannot publish previously copyrighted maps or satellite images created using proprietary data, such as Google software (Google Maps, Street View, and Earth). For more information, see our copyright guidelines: http://journals.plos.org/plosone/s/licenses-and-copyright.

We require you to either (1) present written permission from the copyright holder to publish these figures specifically under the CC BY 4.0 license, or (2) remove the figures from your submission:

**Response:** Thank you. We are now permitted to publish the maps produced from the shapefiles downloaded from DIVA-GIS database (https://www.diva-gis.org/) under a CC BY license, with permission from Global Administrative Areas (GADM). The written permission granted by Prof Robert Hijmans, owner GADM, is included in the manuscript submission as a Supporting Information file.

1. You may seek permission from the original copyright holder of Figure(s) [1, 3, 4, 7 and 8] to publish the content specifically under the CC BY 4.0 license.

**Response:** Thank you. We are now permitted to publish the maps produced from the shapefiles downloaded from DIVA-GIS database (https://www.diva-gis.org/) under a CC BY license, with permission from Global Administrative Areas (GADM). The written permission granted by Prof Robert Hijmans, owner GADM, is included in the manuscript submission as a Supporting Information file.

We recommend that you contact the original copyright holder with the Content Permission Form (http://journals.plos.org/plosone/s/file?id=7c09/content-permission-form.pdf) and the following text:

“I request permission for the open-access journal PLOS ONE to publish XXX under the Creative Commons Attribution License (CCAL) CC BY 4.0 (http://creativecommons.org/licenses/by/4.0/). Please be aware that this license allows unrestricted use and distribution, even commercially, by third parties. Please reply and provide explicit written permission to publish XXX under a CC BY license and complete the attached form.”

**Response:** Thank you. We are now permitted to publish the maps produced from the shapefiles downloaded from DIVA-GIS database (https://www.diva-gis.org/) under a CC BY license, with permission from Global Administrative Areas (GADM). The written permission granted by Prof Robert Hijmans, owner GADM, is included in the manuscript submission as a Supporting Information file.

Please upload the completed Content Permission Form or other proof of granted permissions as an "Other" file with your submission.

**Response:** Thank you. We are now permitted to publish the maps produced from the shapefiles downloaded from DIVA-GIS database (https://www.diva-gis.org/) under a CC BY license, with permission from Global Administrative Areas (GADM). The written permission granted by Prof Robert Hijmans, owner GADM, is included in the manuscript submission as a Supporting Information file.

In the figure caption of the copyrighted figure, please include the following text: “Reprinted from [ref] under a CC BY license, with permission from [name of publisher], original copyright [original copyright year].”

**Response:** Thank you. We are now permitted to publish the maps produced from the shapefiles downloaded from DIVA-GIS database (https://www.diva-gis.org/) under a CC BY license, with permission from Global Administrative Areas (GADM). The written permission granted by Prof Robert Hijmans, owner GADM, is included in the manuscript submission as a Supporting Information file.

2. If you are unable to obtain permission from the original copyright holder to publish these figures under the CC BY 4.0 license or if the copyright holder’s requirements are incompatible with the CC BY 4.0 license, please either i) remove the figure or ii) supply a replacement figure that complies with the CC BY 4.0 license. Please check copyright information on all replacement figures and update the figure caption with source information. If applicable, please specify in the figure caption text when a figure is similar but not identical to the original image and is therefore for illustrative purposes only.

The following resources for replacing copyrighted map figures may be helpful:

USGS National Map Viewer (public domain): http://viewer.nationalmap.gov/viewer/

The Gateway to Astronaut Photography of Earth (public domain): http://eol.jsc.nasa.gov/sseop/clickmap/

Maps at the CIA (public domain): https://www.cia.gov/library/publications/the-world-factbook/index.html and https://www.cia.gov/library/publications/cia-maps-publications/index.html

NASA Earth Observatory (public domain): http://earthobservatory.nasa.gov/

Landsat: http://landsat.visibleearth.nasa.gov/

USGS EROS (Earth Resources Observatory and Science (EROS) Center) (public domain): http://eros.usgs.gov/#

Natural Earth (public domain): http://www.naturalearthdata.com/

**Response:** Thank you. We are now permitted to publish the maps produced from the shapefiles downloaded from DIVA-GIS database (https://www.diva-gis.org/) under a CC BY license, with permission from Global Administrative Areas (GADM). The written permission granted by Prof Robert Hijmans, owner GADM, is included in the manuscript submission as a Supporting Information file.

[Note: HTML markup is below. Please do not edit.]

Reviewers' comments:

Reviewer's Responses to Questions

Comments to the Author

1. Is the manuscript technically sound, and do the data support the conclusions?

The manuscript must describe a technically sound piece of scientific research with data that supports the conclusions. Experiments must have been conducted rigorously, with appropriate controls, replication, and sample sizes. The conclusions must be drawn appropriately based on the data presented.

Reviewer #1: Yes

Reviewer #2: Yes

2. Has the statistical analysis been performed appropriately and rigorously?

Reviewer #1: Yes

Reviewer #2: I Don't Know

3. Have the authors made all data underlying the findings in their manuscript fully available?

The PLOS Data policy requires authors to make all data underlying the findings described in their manuscript fully available without restriction, with rare exception (please refer to the Data Availability Statement in the manuscript PDF file). The data should be provided as part of the manuscript or its supporting information or deposited to a public repository. For example, in addition to summary statistics, the data points behind means, medians and variance measures should be available. If there are restrictions on publicly sharing data—e.g. participant privacy or use of data from a third party—those must be specified.

Reviewer #1: Yes

Reviewer #2: Yes

4. Is the manuscript presented in an intelligible fashion and written in standard English?

PLOS ONE does not copyedit accepted manuscripts, so the language in submitted articles must be clear, correct, and unambiguous. Any typographical or grammatical errors should be corrected at revision, so please note any specific errors here.

Reviewer #1: Yes

Reviewer #2: Yes

5. Review Comments to the Author

Please use the space provided to explain your answers to the questions above. You may also include additional comments for the author, including concerns about dual publication, research ethics, or publication ethics. (Please upload your review as an attachment if it exceeds 20,000 characters)

Reviewer #1: Generally:

1. what is the reason combined different year data set?

**Response:** Thank you. Please see response in ‘General Comments’ above.

2.why not use the informative prior?

**Response:** Thank you. Please see response in ‘General Comments’ above.

3.how to handle the missing values? please explain the algorithm to solve these this problems.

**Response:** Thank you. Please see response in ‘General Comments’ above.

4. why not separate these discussion and conclusion?

**Response:** Thank you. Please see response in ‘General Comments’ above.

Reviewer #2: • Well written

• Please refer to the Fig 1. The labelling of the diagram is wrong as the States in the South East of Nigeria were labelled as South South and vice versa.

**Response:** Thank you. This has now been corrected.

• Kindly go through all the statistical reports to be sure there is no mix-up of data reported for the various geographical zones and if any, please make appropriate corrections throughout the manuscript.

**Response:** Thank you. No such mix-up in data/results reported was identified.

6. PLOS authors have the option to publish the peer review history of their article (what does this mean?). If published, this will include your full peer review and any attached files.

If you choose “no”, your identity will remain anonymous, but your review may still be made public.

Do you want your identity to be public for this peer review? For information about this choice, including consent withdrawal, please see our Privacy Policy.

Reviewer #1: No

Reviewer #2: No

[NOTE: If reviewer comments were submitted as an attachment file, they will be attached to this email and accessible via the submission site. Please log into your account, locate the manuscript record, and check for the action link "View Attachments". If this link does not appear, there are no attachment files.]

While revising your submission, please upload your figure files to the Preflight Analysis and Conversion Engine (PACE) digital diagnostic tool, https://pacev2.apexcovantage.com/. PACE helps ensure that figures meet PLOS requirements. To use PACE, you must first register as a user. Registration is free. Then, login and navigate to the UPLOAD tab, where you will find detailed instructions on how to use the tool. If you encounter any issues or have any questions when using PACE, please email PLOS at figures@plos.org. Please note that Supporting Information files do not need this step.

**Response:** Thank you very much.
